# Disparities in the Prevalence and Risk Factors for Carotid and Lower Extremities Atherosclerosis in a General Population—Bialystok PLUS Study

**DOI:** 10.3390/jcm12072627

**Published:** 2023-03-31

**Authors:** Anna Lisowska, Marlena Dubatówka, Małgorzata Chlabicz, Jacek Jamiołkowski, Marcin Kondraciuk, Anna Szyszkowska, Małgorzata Knapp, Anna Szpakowicz, Adam Łukasiewicz, Karol Kamiński

**Affiliations:** 1 Department of Cardiology, Medical University of Bialystok, 15-276 Bialystok, Poland; 2 Department of Population Medicine and Lifestyle Diseases Prevention, Medical University of Bialystok, 15-276 Bialystok, Poland; 3 Department of Invasive Cardiology, Medical University of Bialystok, 15-276 Bialystok, Poland; 4 Department of Radiology, Medical University of Bialystok, 15-276 Bialystok, Poland

**Keywords:** atherosclerosis, risk factors, lower extremities atherosclerotic disease, carotid atherosclerosis

## Abstract

This study was conducted in a representative sample of area residents aged 20–80 years old. The aim of the study was to assess the prevalence of classic risk factors of atherosclerosis in the studied population and to search for new risk factors in these patient subpopulations. A total of 795 people (mean age 48.64 ± 15.24 years, 45.5% male) were included in the study group. Two independent data analyses were performed. In the first analysis, the study group was divided into two subgroups depending on the presence or absence of atherosclerotic plaques in carotid arteries (APCA). APCA were observed in 49.7% of the study group: in the population aged between 41 and 60 years in 49.3%, and those between 61 and 70 years in 86.3%. Patients with APCA were more often diagnosed with arterial hypertension, diabetes, and hypercholesterolemia. In the second analysis, the study group was divided into two subgroups depending on the presence of lower extremities atherosclerotic disease (LEAD). Patients with an ABI (ankle-brachial index) ≤ 0.9 constituted 8.5% of the study group, and they were significantly older, and more often diagnosed with diabetes and APCA. To identify the factors most strongly associated with APCA and an ABI ≤ 0.9, logistic regression was used, with stepwise elimination of variables. The strongest factors associated with APCA were current smoking and diastolic central pressure. We did not note such an association and did not find additional parameters to facilitate the diagnosis of LEAD in asymptomatic patients. The most important observation in our study was the high prevalence of APCA in the study population, especially in the group of young people under the age of 60.

## 1. Introduction

Atherosclerosis is a systemic disease that affects the arteries of various vascular beds. Patients with atherosclerosis are at elevated risk of ischemic events depending on their specific manifestations. Patients with peripheral artery disease—carotid atherosclerosis (CA) or the lower extremities atherosclerotic disease (LEAD)—are at high risk for major adverse cardiovascular events [1,2,3], and those last-mentioned are also at high risk of major adverse limb events such as severe limb ischemia and amputation [4]. In contrast, patients with CA are at risk of stroke, which is a leading cause of death and of disability-adjusted years of life lost [5]. Also very important is the fact that most strokes (77%) are first-ever strokes, underscoring the importance of effective primary prevention strategies [6]. However, still, despite the use of effective secondary prevention strategies, 5% to 10% of patients with cardiovascular disease have recurrent events each year [2], and cardiovascular diseases remain the leading cause of death in Europe.

Therefore, the knowledge of risk factors for ischemic events in a wide population of patients with atherosclerosis, often still clinically asymptomatic, is crucial and extremely useful for clinical purposes. The ability to promptly identify these risk factors is not only essential in daily clinical practice but would also be useful to investigate novel preventive therapies in patients at particularly high cardiovascular risk. Most of the studies conducted so far in this field concerned patients with symptomatic atherosclerosis and/or clinically asymptomatic, but with numerous known cardiovascular risk factors [2,7]. In contrast, our study was population-based and included randomly selected individuals aged 20–80 years old living in a selected administrative region of the country. The aim of the study was, first, to assess the prevalence of classic risk factors of atherosclerosis in the studied population, including factors specific for atherosclerotic disease of the carotid and lower-limb arteries, and second, to search for new atherosclerosis risk factors in these patient subpopulations.

## 2. Materials and Methods

### 2.1. Study Population

The study was conducted in a representative sample of area residents aged 20–80 years old. Randomly chosen from the mayor’s office database, 1847 residents were invited in 2018–2020 to participate in the study. Altogether, 797 individuals accepted the invitation and were examined. Due to incomplete ultrasound data, 2 people were excluded from further analysis. As a result, 795 people (mean age 48.64 ± 15.24 years, 45.5% male) were included in the study group.

### 2.2. The Estimation of the Number of the Local Inhabitants

Estimation of the number of residents who may have atherosclerotic plaques in the carotid arteries or an ankle-brachial index (ABI) ≤ 0.9 was performed as follows. We obtained the prevalence of atherosclerotic plaques or an ABI ≤ 0.9 in the study population (n = 795) and based on the results, we recalculated the prevalence of atherosclerotic plaques in carotid arteries (APCA) or an ABI ≤ 0.9 in the entire city population using 5-year strata, separately for men and women. In 2022, the city population aged 20–79 was 201,789, including 108,940 females. After applying the above conversion method using 5-year strata, it was estimated that there were 93,842 residents with APCA and 17,264 with an ABI < 0.9 in the age group included in our study (Table 1).

### 2.3. Data Collection

A thorough medical history, family medical history, demographics, and lifestyle data were collected from all participants at the time of the study entry. All subjects underwent medical examination and blood was taken for laboratory evaluation. Peripheral blood was collected in the morning after being fasted for a minimum of 8 h.

Height and weight were measured and body mass index (BMI), as weight (kg)/height (m)^2^, and body surface area (BSA), as (weight (kg) − 60) × 0.01 + height (m) [8], were calculated. Based on the measurement of the waist and hip circumference, the waist-to-hip ratio (WHR) was calculated. Body composition was evaluated by dual-energy X-ray absorptiometry (DEXA) (GE Healthcare, Chicago, IL, USA). The device automatically calculates the amount of android and gynoid adipose tissue. The oscillometric method was used to evaluate artery stiffness parameters, i.e., ankle-brachial index (ABI), brachial-ankle pulse wave velocity (PWVba), and central pressure (CP) (Vascular Explorer, Enverdis, Jena, Germany), in the supine position and preceded by a 10-min rest. Pathological ABI was defined as ≤0.9, measured on at least one side. Blood pressure (BP) was measured by the oscillometric method (Omron Healthcare Co., Ltd. (Kyoto, Japan) MG Comfort) in a sitting position, after a 5-min rest period. For the purpose of this study, hypertension was defined as systolic blood pressure ≥ 140 mm Hg, or diastolic blood pressure ≥ 90 mm Hg, or the history of hypertension, or use of antihypertensive agents.

Ultrasound examinations (echocardiography and ultrasound of the carotid arteries) were performed using ultrasound Vivid 9 (GE Healthcare, Chicago, IL, USA). Heart dimensions in systole (s) and diastole (d) and left ventricular ejection fraction (EF) using the biplane method were taken. The left atrial volume index (LAVI) was calculated, and atrial enlargement was defined as >34 mL/m^2^. The left ventricular mass index (LVMI) was used to define left ventricular hypertrophy (LVH) as LVMI ≥ 95 g/m^2^ for women and ≥115 g/m^2^ for men [9]. Left ventricular diastolic dysfunction was assessed according to the algorithm proposed by EACVI/ASE in 2016 [10]. Those with ‘indeterminate’ diastolic dysfunction were included in the ‘abnormal’ group. The carotid arteries were examined in the supine position. The intima-media thickness (IMT) measurement of the common carotid arteries (CCA) was taken approximately 1 cm before the bifurcation, 5 times per side, and the results were averaged. All measurements were performed on the posterior wall. Plaque was defined according to the Mannheim Intima-Media Thickness Consensus [11] as a focal structure which occupies the lumen of the artery at 0.5 mm or 50% of the surrounding IMT, or is ≥1.5 mm.

The concentrations of N-terminal pro-brain natriuretic peptide (NT-proBNP) and highly sensitive troponin T were determined by the electrochemiluminescence method on the Cobas E411 (ROCHE Diagnostics International Ltd., Rotkreuz, Switzerland). The lipid profile was determined on the Cobas C111 (ROCHE) using the enzymatic-colorimetric method. Glucose concentration and high-sensitivity C-reactive protein (hs-CRP) were marked by the enzymatic method with hexokinase on the same device. For the determination of insulin, we used manual kits from DiaSource with the immunoradiometric method (IRMA). Homeostatic model assessment for insulin resistance (HOMA-IR) was calculated from the following formula: score = fasting insulin (mU/mL) × fasting glucose (mmol/L)/22.5 [12]. For the purposes of the study, diabetes was defined as fasting glucose ≥ 5.6 mmol/L or glucose after 120 min of an oral glucose tolerance test (OGTT) ≥ 11.1 mmol/L, or history of diabetes or use of hypoglycemic agents. Hypercholesterolemia was defined as total cholesterol ≥ 190 mmol/L and/or low-density lipoprotein cholesterol (LDL-cholesterol) ≥ 115 mmol/L, or history of hypercholesterolemia or drugs lowering serum lipid levels. For NTproBNP, the cut-off value was >125 (pg/mL), and for the glomerular filtration rate (GFR), <60 mL/min/m^2^.

### 2.4. Statistical Analysis

The normality test of the Shapiro–Wilk distribution was performed. Descriptive statistics for quantitative variables were presented as median with interquartile range ((IQR) 1st quartile–3rd quartile) and as counts and frequencies for qualitative variables. Comparisons of variables between subgroups were conducted using the Mann–Whitney or Fisher’s tests. Associations between the presence of APCA or ABI and other clinical and biochemical variables were analyzed using a multiple and stepwise backward logistic regression model adjusted for age, gender, hypercholesterolemia, diabetes, hypertension, history of myocardial infarction, and stroke. Statistical hypotheses were verified at the 0.05 significance level. IBM SPSS Statistics 26.0 statistical software (Armonk, NY, USA) was used for all calculations.

The study was conducted in accordance with the Declaration of Helsinki and approved by the Ethics Committee of the Medical University of Bialystok (Poland) (approval number: R-I-002/108/2016, 31 March 2016).

## 3. Results

The number of people with APCA and an ABI ≤ 0.9 in the local population was estimated (Table 1). In the youngest age group of 20–39 years, the prevalence of APCA was estimated at 4021 residents. Among those aged 40 to 59 years, the prevalence of APCA was estimated at 34,977, and in the oldest age group at 23,675. The prevalence of ABI ≤ 0.9 in the 20–39 age group was estimated at 4036 and in the 40–59 age group at 5656. Among those aged 60–69 and 70–79 years, ABI ≤ 0.9 was estimated at 2823 and 4749, respectively. In each age group, it can be noted that the estimated prevalence of an ABI ≤ 0.9 was higher in women.

### 3.1. Carotid Atherosclerosis

**Figure 1:**
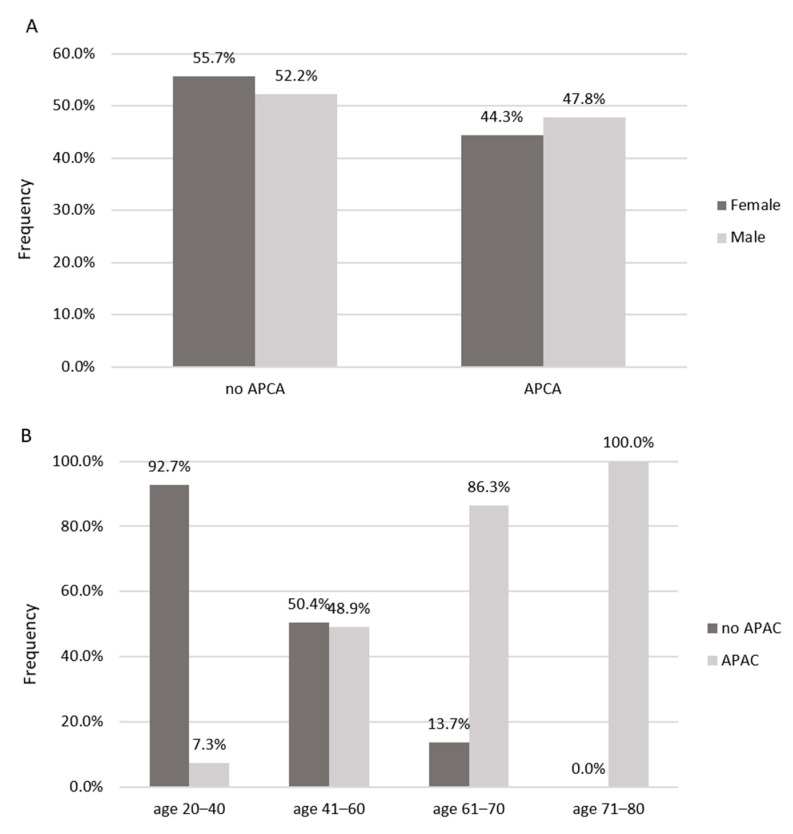
Prevalence of atherosclerotic plaques in carotid arteries by gender (**A**) and age groups (**B**).

The study group (n = 795) was divided into 2 subgroups depending on the presence or absence of APCA ultrasound, and the characteristics of these subgroups are presented in Figure 1 and Table 2.

#### 3.1.1. Demographic Parameters, Interview Data

APCA were observed in 49.7% of the study group: in the population aged between 41 and 60 years in 49.3%, and in those aged 61–70 years in 86.3% (Figure 1B). They were significantly older, and more often diagnosed with arterial hypertension, diabetes, and hypercholesterolemia. This phenomenon was observed more frequently in men. In this subgroup of patients, a history of myocardial infarction or stroke was observed significantly more often. Patients with APCA more often reported reduced tolerance to exercise, exercise dyspnea, and swelling of the lower limbs (Table 2). It was observed that primary and secondary education had a significant impact on the more frequent occurrence of APCA; in people with higher education, APCA were found significantly less often. In the physical examination in this group of patients, significantly higher BP values of both systolic and diastolic as well as increased PWV values were found, but there were no significant differences in ABI values. Patients with diagnosed APCA had a significantly higher BMI and WHR and a significantly higher ratio of android-to-gynoid adipose tissue.

#### 3.1.2. Biochemical Parameters

Patients with APCA had significantly higher fasting glucose levels above normal (≥5.6 mmol/L), consistent with abnormal fasting glycemia, and significantly higher glycemia 2 h after glucose load. Hyperglycemia ≥ 11.1 mmol/L was significantly more frequent in this subgroup of patients (28 (75.7%) vs. 9 (24.3%), *p* < 0.001), which required further diagnosis of de novo diabetes. Additionally, both fasting and 2 h after glucose load insulin and C-peptide levels were significantly higher in patients with APCA.

Significantly higher levels of total cholesterol, LDL cholesterol, and triglycerides were found in this group, with the concentration of LDL exceeding the normal range for the population of healthy people (3.41 (2.61–4.04) mmol/L, with normal up to 3.0 mmol/L). Statistically significant higher levels of hs-CRP, interleukin-6 (Il-6), and a higher percentage of hemoglobin A1c (HbA1c) were also observed. However, there was no statistically significant difference in the concentration of adiponectin between the two studied groups.

A significantly lower GFR was observed in this group, with GFR < 60 mL/min/m^2^ being significantly more frequent (16 patients vs. 0 patients, *p* < 0.001). In subjects without atherosclerotic lesions in the carotid arteries, no impairment of the glomerular filtration rate was observed at all. Significantly higher levels of NTproBNP were observed in the group of patients with APCA—as many as 77% (n = 112) of patients had NTproBNP above normal (>125 pg/mL), while in those without carotid atherosclerosis, only 22.8% (n = 33), *p* < 0.001.

#### 3.1.3. Echocardiographic Examination

Considering the echocardiographic findings, in the group of patients with APCA, a significantly increased left ventricular muscle thickness (interventricular septum (IVS), posterior wall (PW)), larger size of the left atrium, LAVI, and a significantly lower LVEF were found. However, there were no significant differences in the LV dimension between both groups. Left ventricular diastolic dysfunction was significantly more common in patients with APCA.

#### 3.1.4. Pharmacotherapy

Patients with APCA were significantly more likely to use pharmacological treatments, and this applied to all groups of drugs included in the analysis (detailed data in Table 2). However, despite the high prevalence of hypertension in the study group (64.4% of patients with carotid atherosclerosis), only 48.9% of patients took beta-blockers, 51.5% took angiotensin-converting enzyme inhibitors (ACE-inhibitors) or sartans, and 39.1% took calcium channel blockers. An additional analysis among people with hypertension provides more detail on the frequency of medication use (Table S1).

**Figure 2:**
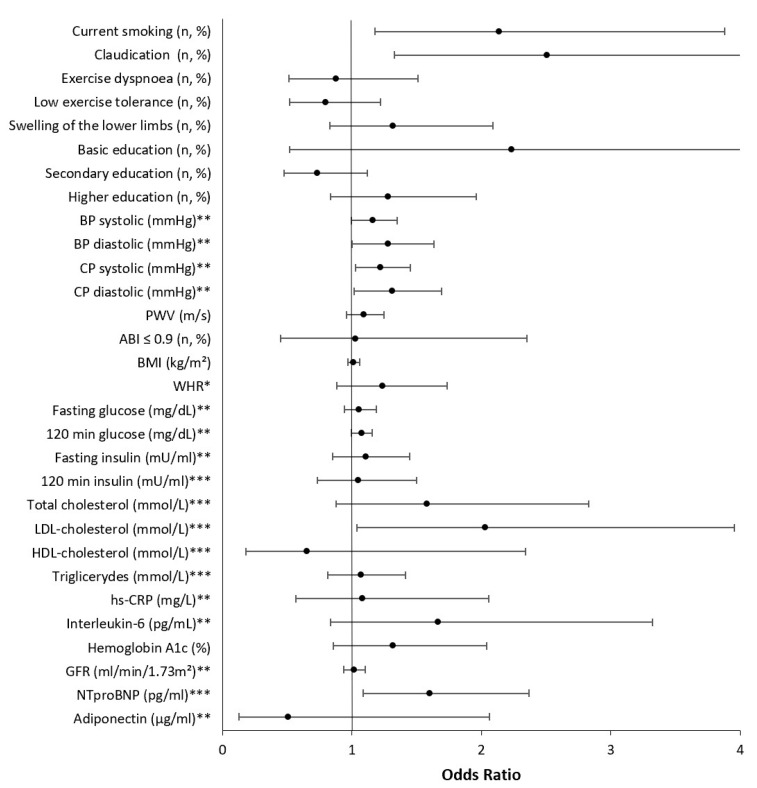
Predictors of APCA in a model adjusted for age, gender, hypercholesterolemia, diabetes, hypertension, history of myocardial infarction, and stroke. * Per 0.1 units; ** per 10 units; *** per 100 units. BP, blood pressure; CP, central pressure; PWV, pulse wave velocity; ABI, ankle-brachial index; IMT, intima-media thickness; BMI, body mass index; WHR, waist-to-hip ratio; LDL, low-density lipoprotein; HDL, high-density lipoprotein; hs-CRP, high-sensitivity C-reactive protein; GFR, glomerular filtration rate; NTproBNP, n-terminal pro-brain natriuretic peptide.

Figure 2 demonstrates the odds of the presence of APCA adjusted for age, gender, hypercholesterolemia, hypertension, diabetes, history of myocardial infarction, and stroke. More frequent smoking (*p* = 0.012) and claudication (*p* = 0.005) were significantly associated with the incidence of APCA. The presence of APCA also increased with raised NTproBNP levels (*p* = 0.018) and LDL-cholesterol (*p* = 0.036). It was noted that despite the correction for hypertension, the increases in systolic (*p* = 0.024) and diastolic CP (*p* = 0.039) were still significantly associated with APCA.

In the logistic regression analysis with stepwise elimination of variables listed in Table 3, the strongest factors associated with APCA were current smoking (*p* = 0.012) and diastolic central pressure (*p* = 0.033).

### 3.2. Lower Extremities Atherosclerosis

**Figure 3:**
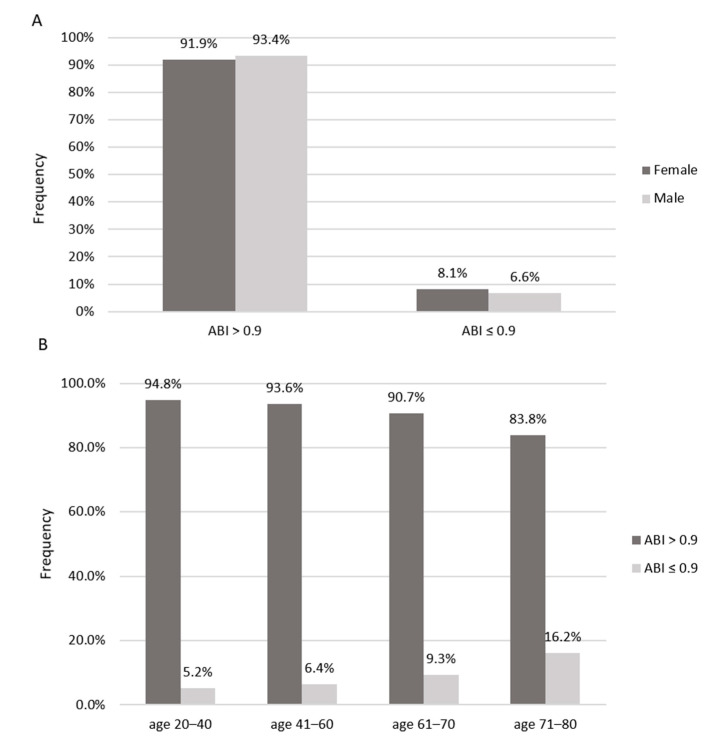
Prevalence of an ABI ≤ 0.9 by gender (**A**) and age groups (**B**).

In the second analysis, the study group (n = 698) was divided into 2 subgroups depending on the presence of lower extremities atherosclerotic disease (LEAD), diagnosed based on the ABI value. An ABI value ≤ 0.9 was considered abnormal according to the ESC and ESVS guidelines [13]. The characteristics of these subgroups are presented in Figure 3 and Table 4.

#### 3.2.1. Demographic Parameters, Interview Data

Patients with an ABI ≤ 0.9 constituted 8.5% of the study group, and they were significantly older, and more often diagnosed with diabetes, APCA, and with a history of stroke. However, the presence of arterial hypertension and hypercholesterolemia or current smoking were not differentiating factors between the two subgroups.

Significantly more patients with an ABI ≤ 0.9 complained of lower extremity pain and swelling compared with the healthy population. A below-normal ABI was found more frequently in those with primary education. On physical examination, there were no significant differences between the two subgroups in blood pressure, BMI, WHR, and PWV. The android/gynoid (A/G) fat ratio was also similar.

#### 3.2.2. Biochemical Parameters

Patients with an ABI ≤ 0.9 had significantly higher fasting and 120 min after the oral loading test glucose levels above the normal values. There were no differences in the concentrations of insulin and C-peptide. However, the percentage of HbA1c was significantly elevated in this group of patients.

There were no significant differences in the concentration of total cholesterol and its fraction, as well as triglycerides and adiponectin in the studied subgroups. Additionally, these patients did not differ significantly in terms of hs-CRP and interleukin-6 levels. In the subgroup of patients with an ABI ≤ 0.9, significantly lower GFR and significantly higher NTproBNP concentrations were observed.

#### 3.2.3. Echocardiographic Examination

Taking into account the results of echocardiography, there were no statistically significant differences in the echocardiographic parameters listed in Table 4 between the analyzed patient subgroups.

#### 3.2.4. Pharmacotherapy

Patients with an ABI ≤ 0.9 were significantly more frequently taking beta-blockers. There were no significant differences in the prevalence of other drugs, i.e., calcium channel blockers, ACE-inhibitors and sartans, diuretics, statins, and antidiabetic drugs.

**Figure 4:**
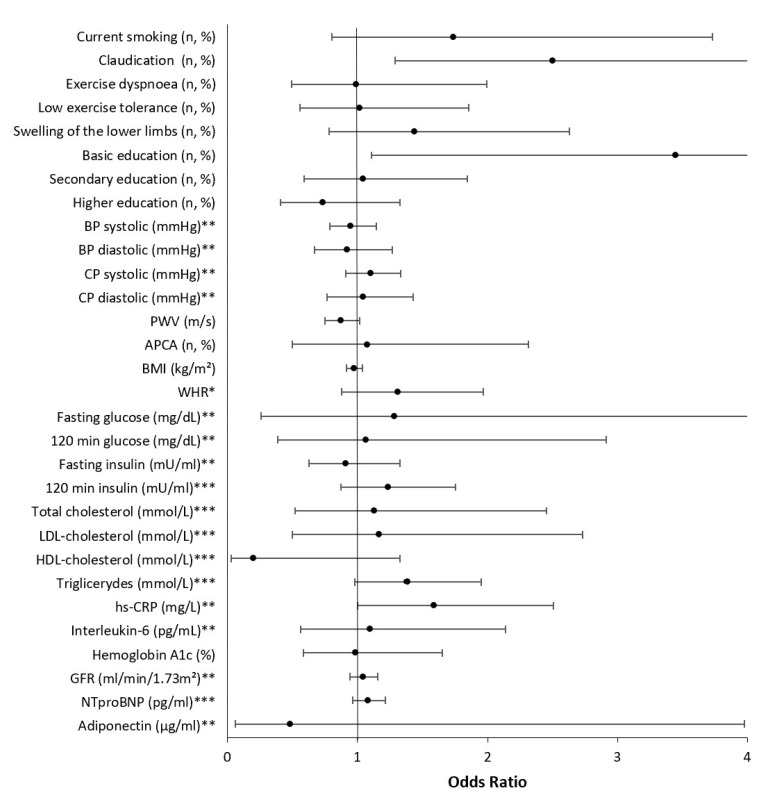
Multivariable predictors of an ABI ≤ 0.9 adjusted for age, gender, hypercholesterolemia, diabetes, hypertension, history of myocardial infarction, and stroke. * Per 0.1 units; ** per 10 units; *** per 100 units. BP, blood pressure; CP, central pressure; PWV, pulse wave velocity; APCA, atherosclerotic plaques in carotid arteries; IMT, intima-media thickness; BMI, body mass index; WHR, waist-to-hip ratio; LDL, low-density lipoprotein; HDL, high-density lipoprotein; hs-CRP, high-sensitivity C-reactive protein; GFR, glomerular filtration rate; NTproBNP, n-terminal pro-brain natriuretic peptide.

Figure 4 shows the adjusted odds of having an ABI ≤ 0.9. In this subpopulation, basic education (*p* = 0.032) was significantly associated with an ABI ≤ 0.9. Claudication (*p* = 0.006) was reported more frequently, and a higher inflammation marker—hs-CPR (*p* = 0.049)—was significantly associated with the presence of an ABI ≤ 0.9.

Logistic regression analysis with stepwise variable elimination was performed to detect which factors are uniquely associated with an ABI ≤ 0.9. Claudication was shown to be independently linked to ABI ≤ 0.9 after accounting for classic cardiovascular factors (age, gender, hypercholesterolemia, diabetes, hypertension, history of myocardial infarction, and stroke) (Table 5).

## 4. Discussion

The Bialystok PLUS study was population-based and included randomly selected people aged 20–80 years living in a specific administrative region of the country. The patients were analyzed depending on the presence of atherosclerotic lesions in peripheral arteries: carotid or lower-limb arteries. Carotid atherosclerosis was diagnosed in the presence of atherosclerotic plaques in the common, bifurcation, external, and/or internal carotid artery. In this group of patients, the classic cardiovascular risk factors, i.e., hypertension, diabetes, hypercholesterolemia, and obesity, were more common, which is consistent with the results of previous studies [1]. The prevalence of atherosclerotic lesions in the carotid arteries was 49.7% in our study, whereas the authors of [14], in a meta-analysis including dozens of articles on the population aged 30–79 years in 2020, estimated the prevalence of atherosclerotic plaques in the carotid arteries at 21.1% (13.2–31.5%). In contrast, in the CSPP study (the China National Stroke Prevention Project) [15], covering the Chinese population in 2014–2015, the incidence of carotid atherosclerosis was 36.2%. It is interesting to note that in our study group, we showed a high percentage of APCA, especially in the young population between the ages of 41 and 60. All patients aged over 70 years had atherosclerotic plaques in the carotid arteries. On the basis of previously published analyses of the Bialystok PLUS study population, it can be assumed that the surprisingly high prevalence of carotid atherosclerosis in our population may be due to the fact that residents have a lower awareness of cardiovascular risk factors and are also less likely to follow preventive recommendations, especially regarding diet and physical activity, in their daily lives [16,17]. Other Polish population-based studies also confirmed that men, less educated persons, and persons with a worse health status were characterized by worse health knowledge [18]. Cigarette smoking was found to be a significant risk factor for carotid atherosclerosis after accounting for numerous classical risk factors, an observation that was similar to the results obtained in another large population-based study conducted in China [19]. Among the new risk factors studied, diastolic central pressure proved to be an independent predictor of the presence of APCA.

It should also be noted that in the group of patients with atherosclerotic plaques in the carotid arteries, a number of abnormalities in biochemical tests were found significantly more often: elevated levels of not only glucose, insulin, C-peptide, and cholesterol, but also CRP and interleukin-6. However, multivariate analysis did not show an effect of inflammatory markers on the presence of atherosclerotic plaques in carotid arteries in the study population. This is in contrast to studies by other authors, who have shown that elevated CRP levels are associated with significantly increased maximum vessel wall thickness in carotid arteries, independent of visceral obesity [20]. Moreover, the role of natriuretic peptide B in the development of atherosclerosis is not clear. In our study, people with confirmed carotid atherosclerosis as well as people with an ABI ≤ 0.9 had significantly elevated NTproBNP levels. However, other authors demonstrated that the plasma NT-proBNP level was not associated with carotid or peripheral atherosclerosis in asymptomatic type 2 diabetic patients with microalbuminuria [21]. These patients also had significantly lower GFR. It is known that a high prevalence of atherosclerotic lesions characterizes patients with chronic kidney disease, though there is little data on the relationship between kidney function and atherosclerotic changes in the healthy population or in people with no known renal impairment. Buscemi et al. showed that the IMT mean values and the prevalence of carotid plaques decreased with the increasing tertile of GFR [22]. Other researchers also observed that the GFR values in the group of patients with coronary artery disease significantly negatively correlated with IMT complex in carotid arteries [23]. Thus, even mildly impaired renal function may be a risk factor for carotid atherosclerosis in asymptomatic individuals and indicates the need for carotid ultrasound screening.

The echocardiographic abnormalities found in this subgroup of patients were primarily due to the high prevalence of hypertension. However, it should be noted that only about 50% of these patients were taking hypotensive medications. It has already been noted that the prevalence and the number of plaques significantly positively related to hypertension categories as well as to the duration of hypertension [24]. Other researchers found carotid subclinical damage in 27.0% of patients with untreated essential hypertension, who were then reclassified in the high-risk stratum [25]. In conclusion, the use of carotid ultrasonography allows a much more accurate identification of high-risk individuals and the application of early, effective primary prevention. Thus, it is surprising that the latest Society for Vascular Surgery guidelines do not recommend carotid ultrasonography for clinically asymptomatic carotid artery stenosis in individuals without cerebrovascular symptoms or significant risk factors for carotid artery disease. However, in selected asymptomatic patients who are at increased risk for carotid stenosis, they suggest screening for clinically asymptomatic carotid artery stenosis [26]. In contrast, the latest ESC 2021 prevention guidelines state that carotid artery plaque assessment using ultrasonography probably also reclassifies cardiovascular disease (CVD) risk and may be considered as a risk modifier in patients at intermediate cardiovascular risk, especially when a coronary artery calcium score is not feasible. Our observations confirm the advisability of carotid screening to search for atherosclerotic plaques in asymptomatic patients. It turns out that these patients are burdened with a much greater number of cardiovascular risk factors and therefore require appropriate education in this area and early application of intensified primary prevention.

Lower extremities atherosclerotic disease (LEAD) is another manifestation of atherosclerotic vascular disease and is also associated with a high cardiovascular risk. Despite its high prevalence, it often remains unrecognized and underdiagnosed. In our study population, we observed a low percentage of patients with an ABI below normal—8.5%. This value is similar to literature data, which showed that the prevalence of LEAD in the population of similar age in high-income countries is on average 7.8%, and in low-income and middle-income countries, 6.4% [27]. On the other hand, in the Palestinian population, LEAD was found in as many as 13.7% of respondents [28]. Currently, the greatest challenge is the identification of individuals with asymptomatic LEAD—in our study population, more than half of the patients with ABI ≤ 0.9 were asymptomatic, which is consistent with previous observations [29]. Unfortunately, in our study, we observed that in multivariate analysis, after taking into account classical risk factors, only claudication was shown to be independently linked to an ABI ≤ 0.9. Thus, our observations did not allow us to find additional parameters to facilitate the diagnosis of LEAD in asymptomatic patients. Moreover, it turned out that diabetes was the most important risk factor associated with the occurrence of LEAD, while no association was found with the occurrence of hypertension or hypercholesterolemia. However, other researchers have found that hypertension is an independent factor in the occurrence of LEAD [28]. Admittedly, a higher prevalence of hypertension and smoking was observed in the study group of LEAD patients, but these differences did not reach statistical significance, most likely due to the small group size. The WOBASZ II study confirmed that in all patients diagnosed with hypertension, in comparison to normotensive subjects, the risk of being diagnosed with PAD was about 50% higher [30].

We observed that the level of education is crucial: people with primary education had significantly higher incidence of atherosclerosis of peripheral arteries, both carotid and femoral, which evidently indicates a low knowledge of cardiovascular risk factors and the necessity of their elimination, but also often limited economic opportunities for prevention. Furthermore, analysis of the group of patients with LEAD revealed that a number of classic and novel cardiovascular risk factors that are relevant in patients with established atherosclerotic plaques in the carotid arteries did not play a significant role in the development of LEAD. In addition, these patients had no significant echocardiographic abnormalities compared with those with normal ABI, which was probably due to the similar prevalence of hypertension and chronic coronary syndrome in both groups.

It has been found that inflammatory parameters—hs-CRP and Il-6—are not useful markers of LEAD. Different results were obtained by other researchers, who observed that elevated levels of interleukin-6 are a risk factor for LEAD [31].

In conclusion, it seems that screening ultrasound examination of the carotid arteries to detect atherosclerotic plaques is justified in a wide population of both asymptomatic individuals and those with single cardiovascular risk factors because it is a good modifier in estimating this risk and thus enables appropriate educational and preventive actions to be taken. In view of the relatively rare below-normal ABI values found in the study population and the lower burden of classical cardiovascular risk factors in these patients, it may be thought that the screening measurement of this index in an asymptomatic population has less clinical significance and gains importance in a population with diabetes or burdened with multiple cardiovascular risk factors.

Some limitations should be noted in the present study. The small number of people with an ABI ≤ 0.9 could have significantly affected the strength of the statistical analysis. Investigations should be carried out in the future after collecting a larger group of participants with lower extremity arteriosclerosis as a study group.

## 5. Conclusions

In our study, we showed a high percentage (49.7%) of atherosclerotic lesions in the carotid arteries in the population of the Bialystok PLUS study, especially in the young population between the ages of 41 and 60. All patients aged over 70 years had atherosclerotic plaques in the carotid arteries. The strongest factors associated with APCA were current smoking and diastolic central pressure. Our observations did not allow us to find additional parameters to facilitate the diagnosis of LEAD in asymptomatic patients.

## Figures and Tables

**Table 1:** Estimated prevalence of atherosclerotic plaques in carotid arteries and an ABI ≤ 0.9 in the local population.

	Total Population		APCA		ABI ≤ 0.9	
	Female	Male	Female	Male	Female	Male
age 20–39	34,140 (16.92%)	33,574 (16.64%)	1048 (0.52%)	2973 (1.47%)	2166 (1.99%)	1870 (1.07%)
age 40–59	39,023 (19.34%)	34,684 (17.19%)	16,321 (8.09%)	18,656 (9.25%)	3025 (17.33%)	2631 (1.5%)
age 60–69	20,844 (10.33%)	15,538 (7.7%)	16,573 (8.21%)	14,596 (7.23%)	2243 (15.45%)	580 (1.11%)
age 71–79	14,933 (7.4%)	9053 (4.49%)	14,622 (7.25%)	9053 (4.49%)	2584 (11.73%)	2165 (1.28%)

Data are presented as an estimated number of residents in a specific age group and percentage. APCA, atherosclerotic plaques in carotid arteries; ABI, ankle-brachial index.

**Table 2:** Characteristics of the study group depending on the presence of atherosclerotic plaques in carotid arteries (APCA).

	Patients without APCA (n = 430)	Patients with APCA (n = 365)	*p*-Value
Age (years)	37.5 (31–45)	62 (53–68)	<0.001
Gender, men (n, %)	189 (44%)	173 (47.4%)	0.353
Current smoking (n,%)	82 (19.1%)	81 (22.2%)	0.556
Hypertension (n, %)	120 (27.9%)	235 (64.4%)	<0.001
Diabetes (n, %)	27 (6.3%)	78 (21.4%)	<0.001
Hypercholesterolemia (n, %)	255 (59.3%)	315 (86.3%)	<0.001
Low exercise tolerance (n, %)	227 (52.8%)	235 (64.4%)	0.001
Exercise dyspnea (n, %)	58 (13.5%)	89 (24.4%)	<0.001
Swelling of the lower limbs (n, %)	104 (24.2%)	163 (44.7%)	<0.001
Claudication (n, %)	24 (5.6%)	85 (23.3%)	<0.001
History of myocardial infarction (n, %)	0.00	13 (3.6%)	<0.001
History of stroke (n, %)	2 (0.5%)	11 (3.0%)	<0.001
Basic education (n, %)	7 (1.6%)	16 (4.4%)	0.032
Secondary education (n, %)	170 (39.5%)	203 (55.6%)	<0.001
Higher education (n, %)	250 (58.1%)	203 (55.6%)	<0.001
BP systolic (mmHg)	119 (108.5–130.5)	129.13 (117–140.94)	<0.001
BP diastolic (mmHg)	79.5 (73–86.5)	82 (76.5–88.5)	<0.001
CP systolic (mmHg)	104 (97–114)	118 (108–129.5)	<0.001
CP diastolic (mmHg)	69 (63.5–76)	75 (69–82)	<0.001
PWV (m/s)	9.7 (8.6–10.8)	11.6 (10.15–13.2)	<0.001
BMI (kg/m^2^)	25.04 (22.47–28.35)	27.85 (24.42–31.46)	<0.001
WHR	0.85 (0.78–0.92)	0.91 (0.84–0.99)	<0.001
A/G fat ratio	0.5 (0.38–0.65)	0.67 (0.5–0.85)	<0.001
ABI ≤ 0.9 (n, %)	25 (5.8%)	34 (9.3%)	0.075
IMT (mm)	0.56 (0.51–0.62)	0.71 (0.63–0.82)	<0.001
Biochemistry			
Fasting glucose (mmol/L)	5.39 (5.05–5.72)	5.77 (5.39–6.22)	<0.001
Glucose 120 min (mmol/L)	6.25 (5.27–7.34)	7.05 (6.16–8.66)	<0.001
Fasting insulin (µUI/mL)	9.79 (7.12–13.78)	11.43 (8–17.01)	<0.001
Insulin 120 min (µUI/mL)	42.77 (26.74–68.17)	54.37 (34.87–92.04)	<0.001
Total cholesterol (mmol/L)	4.73 (4.11–5.33)	5.07 (4.29–5.86)	<0.001
LDL-cholesterol (mmol/L)	3.05 (2.5–3.59)	3.41 (2.61–4.04)	<0.001
HDL-cholesterol (mmol/L)	1.6 (1.34–1.89)	1.54 (1.25–1.85)	0.074
Triglycerides (mmol/L)	0.95 (0.68–1.38)	1.2 (0.91–1.67)	<0.001
hs-CRP (mg/L)	0.58 (0.27–1.3)	0.82 (0.41–1.66)	<0.001
Interleukin-6 (pg/mL)	2.28 (1.83–3.49)	3.08 (2.1–4.23)	<0.001
Hemoglobin A1c (%)	5.3 (5–5.5)	5.6 (5.4–5.9)	<0.001
GFR (ml/min/1.73 m^2^)	113.64 (100.36–128.65)	94.52 (77.9–110.96)	<0.001
NTproBNP (pg/mL)	38.89 (22.55–67.39)	77.6 (38.16–140.9)	<0.001
Troponin T (ng/L)	5.36 (4.42–7.16)	7.15 (5.27–11.03)	<0.001
Adiponectin (µg/mL)	4.57 (3.4–6.22)	4.64 (3.35–6.83)	0.392
Echocardiography			
IVSd (mm)	8.01 (7.24–8.94)	8.87 (8.06–9.88)	<0.001
PWd (mm)	7.65 (6.61–8.59)	8.47 (7.54–9.67)	<0.001
LVMI (g/m^2^)	66.77 (56.44–78)	77.21 (66.85–91.4)	<0.001
LA (mm)	33.33 (30.09–36.69)	35.58 (32.74–39.34)	<0.001
LAVI (mL/m^2^)	20.51 (17.41–24.51)	22.13 (18.3–27.28)	<0.001
LVEF (%)	59.93 (57.16–63.12)	57.74 (54.29–61.74)	<0.001
LVDd (mm)	47.6 (44.33–50.69)	47.46 (44.33–51.82)	0.456
LV diastolic dysfunction (n, %)	12 (2.8%)	15 (14%)	<0.001
Pharmacotherapy			
Beta-blockers	29 (6.7%)	115 (31.5%)	<0.001
Calcium channel blockers	6 (1.4%)	45 (12.3%)	<0.001
ACE-inhibitors and sartans	38 (8.8%)	121 (33.2%)	<0.001
Diuretics	11 (2.6%)	39 (10.7%)	0.019
Statins	17 (4%)	90 (24.7%)	<0.001
Antidiabetic drugs	11 (2.6%)	38 (10.4%)	0.027

Data are presented as median (Q1–Q3) or n (%). BP, blood pressure; CP, central pressure; PWV, pulse wave velocity; BMI, body mass index; WHR, waist-to-hip ratio; A, android; G, gynoid; ABI, ankle-brachial index; IMT, intima-media thickness; LDL, low-density lipoprotein; HDL, high-density lipoprotein; hs-CRP, high-sensitivity C-reactive protein; GFR, glomerular filtration rate; NTproBNP, n-terminal pro-brain natriuretic peptide; IVSd, interventricular septal thickness in diastole; PWd, left ventricle posterior wall thickness in diastole; LVMI, left ventricular mass index; LA, left atrium; LAVI, left atrial volume index; EF, ejection fraction; LVDd, left ventricular dimension in diastole.

**Table 3:** Results of stepwise backward logistic regression analysis of atherosclerotic plaques in carotid arteries.

Variables	Full Model		Final Model	
	*p*-Value	OR (95%CI)	*p*-Value	OR (95%CI)
Current smoking (n, %)	0.009	2.47 (1.25 ± 4.88)	0.012	2.31 (1.21 ± 4.42)
CP diastolic (mmHg)	0.202	1.04 (0.98 ± 1.10)	0.033	1.04 (1.00 ± 1.08)
NTproBNP (pg/mL)	0.097	1.00 (1.00 ± 1.01)	0.090	1.00 (1.00 ± 1.01)
Claudication (n, %)	0.182	1.92 (0.74 ± 4.98)		
LDL-cholesterol (mmol/L)	0.130	1.01 (1.00 ± 1.02)		
CP systolic (mmHg)	0.753	0.99 (0.96 ± 1.06)		
BP diastolic (mmHg)	0.766	1.01 (0.96 ± 1.06)		

Model adjusted for age, gender, hypercholesterolemia, diabetes, hypertension, history of myocardial infarction, and stroke; BP, blood pressure; CP, central pressure; LDL, low-density lipoprotein; NTproBNP: n-terminal pro-brain natriuretic peptide.

**Table 4:** Characteristics of the study group depending on the ABI ≤ 0.9.

	Patients with ABI > 0.9 (n = 639)	Patients with ABI ≤ 0.9 (n = 59)	*p*-Value
Age (years)	46 (36–62)	56 (40–69)	0.012
Gender, men (n, %)	284 (44.4%)	24 (40.7%)	0.681
Current smoking (n, %)	131 (20.5%)	16 (27.1%)	0.128
Hypertension (n, %)	282 (44.1%)	31 (52.5%)	0.274
Diabetes (n, %)	79 (12.4%)	13 (22.0%)	0.043
Hypercholesterolemia (n, %)	454 (71.0%)	43 (72.9%)	0.762
Low exercise tolerance (n, %)	377 (59.0%)	38 (64.4%)	0.579
Exercise dyspnea (n, %)	116 (18.2%)	14 (23.7%)	0.294
Swelling of the lower limbs (n, %)	218 (34.1%)	29 (49.2%)	0.032
Claudication (n, %)	80 (12.5%)	19 (32.2%)	<0.001
History of myocardial infarction (n, %)	10 (1.6%)	2 (3.4%)	0.271
History of stroke (n, %)	6 (0.9%)	3 (5.1%)	0.034
Basic education (n, %)	14 (2.2%)	5 (8.5%)	0.017
Secondary education (n, %)	303 (47.4%)	32 (54.2%)	0.344
Higher education (n, %)	319 (49.9%)	22 (37.3%)	0.076
BP systolic (mmHg)	123.5 (111.5–135.5)	121 (109–136.5)	0.526
BP diastolic (mmHg)	81 (74.5–87.5)	80 (75–85)	0.629
CP systolic (mmHg)	111 (100–122)	112 (103–131)	0.190
CP diastolic (mmHg)	71 (65–79)	73 (66–78)	0.629
PWV (m/s)	10.4 (9.18–11.9)	10.8 (8.5–12)	0.824
BMI (kg/m^2^)	26.14 (23.24–29.62)	26.97 (23.29–30.54)	0.445
WHR	0.87 (0.8–0.95)	0.91 (0.84–0.96)	0.081
A/G fat ratio	0.57 (0.41–0.75)	0.59 (0.45–0.73)	0.467
APCA (n, %)	0.61 (0.53–0.72)	0.63 (0.54–0.76)	0.037
IMT (mm)	123.5 (111.5–135.5)	121 (109–136.5)	0.183
Biochemistry			
Fasting glucose (mmol/L)	5.55 (5.16–5.94)	5.72 (5.33–6.27)	0.035
120 min glucose (mmol/L)	6.66 (5.61–7.83)	6.99 (5.69–9.3)	0.215
Fasting insulin (µUI/mL)	10.24 (7.58–15.56)	10.74 (7.82–14.22)	0.783
120 min insulin (µUI/mL)	48.34 (29.53–75.96)	56.82 (29.18–91.25)	0.249
Total cholesterol (mmol/L)	4.81 (4.15–5.5)	4.63 (4.14–5.88)	0.746
LDL-cholesterol (mmol/L)	3.15 (2.56–3.76)	3.16 (2.42–3.82)	0.702
HDL-cholesterol (mmol/L)	1.55 (1.3–1.86)	1.54 (1.19–1.86)	0.342
Triglycerides (mmol/L)	1.02 (0.77–1.47)	1.2 (0.9–1.75)	0.035
hs-CRP (mg/L)	0.67 (0.32–1.52)	0.7 (0.4–1.71)	0.186
Interleukin-6 (pg/mL)	2.71 (1.92–3.68)	3.03 (2.04–4.89)	0.156
Hemoglobin A1c (%)	5.4 (5.1–5.7)	5.6 (5.2–5.9)	0.020
GFR (ml/min/1.73 m^2^)	106.37 (89.48–122.13)	95.65 (86–114.64)	0.033
NTproBNP (pg/mL)	50.99 (27.6–97.2)	80.77 (39.82–138.55)	0.001
Troponin T (ng/L)	6.03 (4.62–8.47)	6.48 (5.13–9.96)	0.181
Adiponectin (µg/mL)	4.64 (3.38–6.5)	4.51 (3.49–5.22)	0.465
Echocardiography			
IVSd (mm)	8.45 (7.59–9.44)	8.4 (7.42–9.51)	0.955
PWd (mm)	8.06 (7.02–9.16)	8.19 (6.89–9.68)	0.586
LVMI (g/m^2^)	71.63 (60.55–83.49)	71.5 (59.47–84.43)	0.963
LA (mm)	34.07 (31.11–37.86)	33.83 (29.79–38.36)	0.648
LAVI (mL/m^2^)	21.3 (18.02–25.57)	20.81 (16.68–26.34)	0.926
EF Biplane (%)	59.1 (55.75–62.39)	58 (53.83–61.86)	0.082
LVDd (mm)	47.48 (44.45–51.04)	46.21 (42.5–49.84)	0.048
LV diastolic dysfunction (n, %)	49 (7.7%)	8 (13.6%)	0.132
Pharmacotherapy			
Beta-blockers	108 (16.9%)	18 (30.5%)	0.041
Calcium channel blockers	42 (6.6%)	6 (10.2%)	0.443
ACE-inhibitors and sartans	125 (19.6%)	11 (18.6%)	0.466
Diuretics	35 (5.5%)	7 (11.9%)	0.16
Statins	83 (13.0%)	14 (23.7%)	0.075
Antidiabetic drugs	36 (5.6%)	6 (10.2%)	0.275

Data are presented as median (Q1–Q3) or n (%). BP, blood pressure; CP, central pressure; PWV, pulse wave velocity; BMI, body mass index; WHR, waist-to-hip ratio; A, android; G, gynoid; APCA, atherosclerotic plaques in carotid arteries; IMT, intima-media thickness; LDL, low-density lipoprotein; HDL, high-density lipoprotein; hs-CRP, high-sensitivity C-reactive protein; GFR, glomerular filtration rate; NTproBNP, n-terminal pro-brain natriuretic peptide; IVSd, interventricular septal thickness in diastole; PWd, left ventricle posterior wall thickness in diastole; LVMI, left ventricular mass index; LA, left atrium; LAVI, left atrial volume index; EF, ejection fraction; LVDd, left ventricular dimension in diastole.

**Table 5:** Results of stepwise backward logistic regression analysis of an ABI ≤ 0.9.

Variables	Full Model		Final Model	
	*p*-Value	OR (95%CI)	*p*-Value	OR (95%CI)
Claudication (n, %)	0.006	2.46 (1.3 ± 4.64)	0.008	2.32 (1.25 ± 4.32)
Basic education (n, %)	0.312	2.09 (0.5 ± 8.74)		
hs-CRP (mg/L)	0.812	1.01 (0.95 ± 1.08)		

Model adjusted for age, gender, hypercholesterolemia, diabetes, hypertension, history of myocardial infarction, and stroke. hs-CRP, high-sensitivity C-reactive protein.

## Data Availability

Not applicable.

## Supplementary Materials

The following supporting information can be downloaded at: https://www.mdpi.com/article/10.3390/jcm12072627/s1, Table S1: Characteristics of hypertensive subjects in the study population by APCA and ABI ≤ 0.9—medications used.
