# Supplementary material for: Disparities in the Prevalence and Risk Factors for Carotid and Lower Extremities Atherosclerosis in a General Population—Bialystok PLUS Study
Source: J Clin Med. 2023 Mar 31;12(7):2627. doi: 10.3390/jcm12072627 (PMC10095274; doi:10.3390/jcm12072627)
Supplement: Supplementary file 1 [file jcm-12-02627-s001.zip › jcm-2211292-supplementary.pdf]

Table S1. Characteristics of hypertensive subjects in the study population by APCA and ABI ≤ 0.9 - medications used.

	Patients without APCA (n=120)	Patients with APCA (n=235)	P value	Patients with ABI > 0.9 (n=282)	Patients with ABI ≤ 0.9 (n=31)	P value
Beta-blockers	29 (42%)	114 (58.2%)	0.025	107 (52.2%)	18 (69.2%)	0.143
Calcium channel blockers	6 (8.7%)	45 (23%)	0.012	42 (20.5%)	6 (23.1%)	0.798
ACE- inhibitors and sartans	38 (55.1%)	120 (61.2%)	0.394	124 (60.5%)	11 (42.3%)	0.092
Diuretics	11 (15.9%)	39 (19.9%)	0.592	35 (17.1%)	7 (26.9%)	0.277
